# The Treatment of Post-Partum Hemorrhage

**Published:** 1893-09

**Authors:** 


THE TREATMENT OF POST-PARTUM HEMORRHAGE.—Herman (*Revue Medico-Chirurgicale des Maladies, des Femmes*, May 25, 1893,) statesthat compression of the vessels is the most rational means of arresting post-partum hemorrhage. As preventive means the following considerations should be borne in mind: To render assistance if the uterus is inactive; to pay most minute attention to the details of the third portion of delivery. As treatment he advises massage of the uterus, with the hand placed upon the abdomen. If this is not successful, the introduction of the hand into the uterus to prove conclusively that it is perfectly empty. Finally, injections of hot water within the uterine cavity. If these means fail, persistent bimanual compression of the uterus should be made, assisted in its action by putting the infant to the breast. Injections of iron solution are dangerous, because they may cause death by distention of the uterus, peritonitis, or septicæmia. The introduction of iodoform gauze is not without its disadvantages; sometimes it is a means of allowing air to enter the veins, and it always prevents normal contraction of the uterus.—*Therapeutic Gazette.*

